# Dormant cancer cells accumulate high protoporphyrin IX levels and are sensitive to 5-aminolevulinic acid-based photodynamic therapy

**DOI:** 10.1038/srep36478

**Published:** 2016-11-18

**Authors:** Taku Nakayama, Shimpei Otsuka, Tatsuya Kobayashi, Hodaka Okajima, Kentaro Matsumoto, Yuichiro Hagiya, Keiji Inoue, Taro Shuin, Motowo Nakajima, Tohru Tanaka, Shun-ichiro Ogura

**Affiliations:** 1 Graduate School of Bioscience and Biotechnology, Tokyo Institute of Technology, 4259 B47, Nagatsuta-cho, Midori-ku, Yokohama 226-8501, Japan; 2 Department of Urology, Kochi Medical School, Kohasu, Okocho, Nankoku, Kochi, 783-8505, Japan; 3 SBI Pharmaceuticals Co., Ltd., Izumi Garden Tower 20F, 1-6-1, Roppongi, Minato-ku, Tokyo 106-6020, Japan

## Abstract

Photodynamic therapy (PDT) and diagnosis (PDD) using 5-aminolevulinic acid (ALA) to drive the production of an intracellular photosensitizer, protoporphyrin IX (PpIX), are in common clinical use. However, the tendency to accumulate PpIX is not well understood. Patients with cancer can develop recurrent metastatic disease with latency periods. This pause can be explained by cancer dormancy. Here we created uniformly sized PC-3 prostate cancer spheroids using a 3D culture plate (EZSPHERE). We demonstrated that cancer cells exhibited dormancy in a cell density-dependent manner not only in spheroids but also in 2D culture. Dormant cancer cells accumulated high PpIX levels and were sensitive to ALA-PDT. In dormant cancer cells, transporter expressions of PEPT1, ALA importer, and ABCB6, an intermediate porphyrin transporter, were upregulated and that of ABCG2, a PpIX exporter, was downregulated. PpIX accumulation and ALA-PDT cytotoxicity were enhanced by G0/G1-phase arrestors in non-dormant cancer cells. Our results demonstrate that ALA-PDT would be an effective approach for dormant cancer cells and can be enhanced by combining with a cell-growth inhibitor.

Tumor cells accumulate protoporphyrin IX (PpIX) after treatment with 5-aminolevulinic acid (ALA)^1^,^2^,^3^. PpIX fluorescence can be visualized using a modified neurosurgical microscope and is used for photodynamic diagnosis (PDD). This detection is particularly used in glioma, bladder cancer, and prostate cancer^4^,^5^,^6^. Furthermore, PpIX generates singlet oxygen and other reactive oxygen species on visible light irradiation^7^,^8^,^9^. ALA-based photodynamic therapy (ALA-PDT) can lead to cell death via the necrosis or apoptosis pathway and is a highly effective form of therapy for treating superficial basal cell carcinomas.

Although ALA-PDD or -PDT are widespread in clinical use, PpIX accumulation mechanism and the difference between high- and low-accumulation cancer cells remain unclear. In a previous study, we demonstrated that the peptide transporter PEPT1 and the ATP-binding cassette transporter ABCG2 were key players in regulating intracellular PpIX *in vitro* and in bladder cancer specimens^10^. Moreover, we identified the effects of plasma membrane ABCB6 on porphyrin accumulation under hypoxic conditions^11^. In a recent study^12^, ABCB6 upregulation played a key role in PpIX accumulation. Altogether, these results suggest that transporters play a critical role in porphyrin metabolism.

Patients with cancer can develop a recurrent metastatic disease with latency periods that range from years to even decades^13^,^14^. This pause can be explained by cancer dormancy^13^. Dormant cancer cells whose physiological functions pause or become quiescent are relatively insensitive to most chemotherapeutic drugs and radiation. The cells can cause tumor recurrence when they re-enter the cell cyc^13^,^15^,^16^. The mechanism of entering the dormant state is poorly understood; however, dormant cancer cells can be characterized by the absence of proliferation or death, metabolic suppression, and recovery of active status^17^,^18^.

Tumor cells in the human body interact with neighboring cells and are present in a high cell density. However, cancer cells grown in a 2D monolayer culture are at a low density and lose tissue-specific properties^19^,^20^. A 3D-cultured cancer spheroid achieves much higher cell density than 2D culture and reproduces several parameters of the tumor microenvironment, including oxygen and nutrient gradients and the development of dormant tumor regions^15^,^19^,^21^,^22^. Furthermore, high cell density leads to contact inhibition. Since Contact inhibition suppresses cell growth^23^, cancer spheroids possibly enter the dormant state. Dormant cancer cells are insensitive to most treatments, although there are no reports between those cells and ALA-PDT effect. We speculated that porphyrin metabolism after cell dormancy and ALA treatment are simultaneously regulated in cancer spheroids in a 3D culture. In this study, we tested this hypothesis.

## Results

### Cancer spheroids were formed in EZSPHERE 3D culture plates

**Figure 1:**
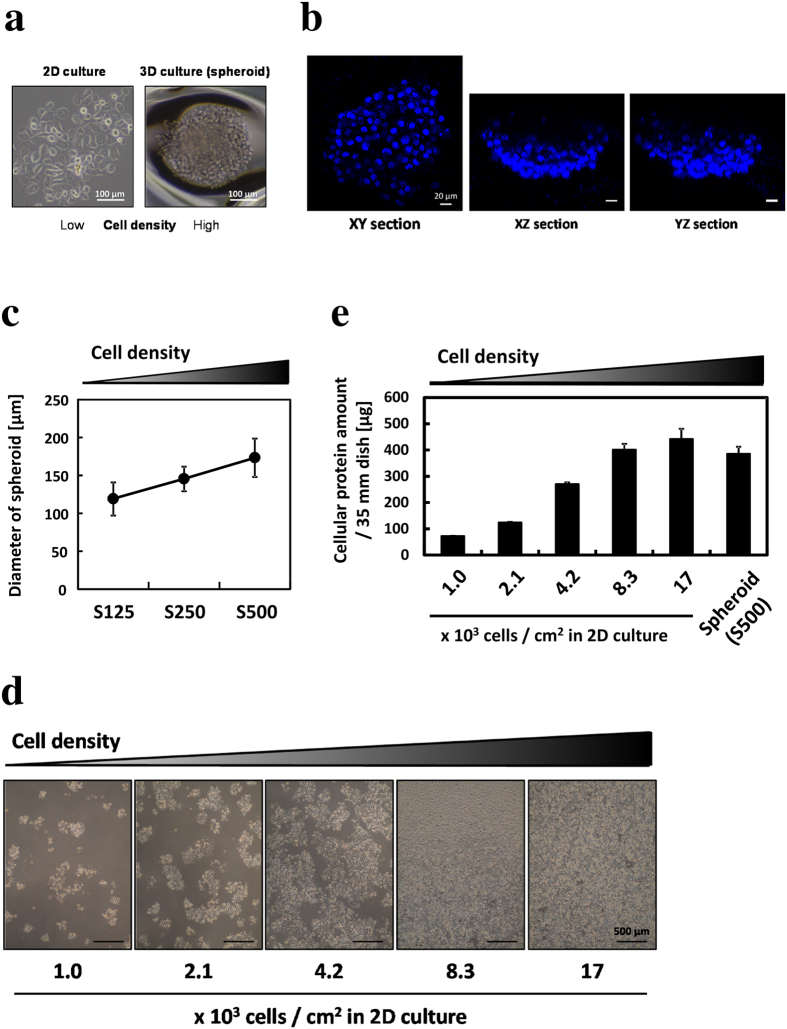
PC-3 spheroid formed in an EZSPHERE 3D culture plate and different cell density in the 2D culture. All cells were incubated for 4 days. (**a**) Phase-contrast images of a spheroid. Scale bar, 100 μm. (**b**) Hoechst 33342 stains for nuclei. A z-stack section was obtained every 2 μm. Scale bar, 20 μm. (**c**) The diameter of formed spheroids. S125, 1.25; S250, 2.50; S500, 5.00 × 10^5^ cells/35-mm dish. Over 40 spheroids were counted. (**d**) Phase-contrast images of cells in the 2D culture. Scale bar, 500 μm. (**e**) Cellular protein amount measured by Bradford assay; n = 3. All bars represent standard deviation (SD).

An aliquot of 5 × 10^5^ PC-3 prostate cancer cells was seeded in every 35-mm dish of the EZSPHERE 3D culture plate. During seeding, the cell number was the same in each microwell of the culture dish. Four days after seeding, approximately 2,300 spheroids had formed in each dish. The average diameter of the spheroids was 189 ± 30.1 μm (Fig. 1a). Hoechst staining of spheroids revealed a cell density higher than that in a 2D culture and a spheroid thickness of approximately 80 μm (Fig. 1b). This thickness is sufficient for oxygen diffusion because spheroids above 200 μm in diameter experience hypoxic conditions at the core^22^. Similarly, we formed two other sizes of spheroids by reducing the number of seeded cells (Fig. 1c). To create different cell density conditions in a 2D culture, cells were seeded in every 35-mm flat dish to a specified cell density and cultured for 4 days (Fig. 1d). The cellular protein amount has a positive relationship with cell density (Fig. 1e). We used 3D cultured spheroids and 2D cultured cells for subsequent experiments.

### Cancer cells show dormancy depending on cell density in 2D culture and on spheroid formation

**Figure 2:**
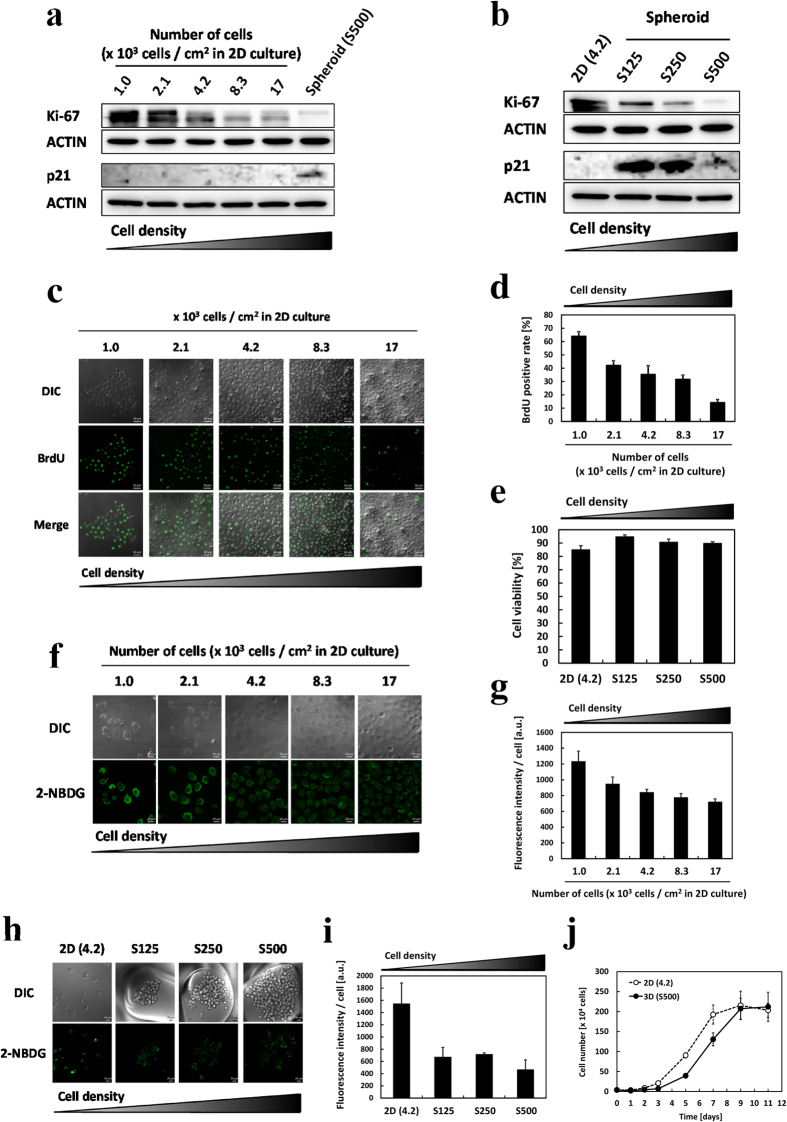
Investigation of cell proliferation, cell death, glucose uptake, and recovery of active status in the 2D culture and spheroids. (**a**) Protein expression level of Ki-67 and p21 in the 2D culture was detected by Western blotting. (**b**) Protein expression levels of Ki-67 and p21 in the 3D culture were detected by Western blotting. (**c**) Immunochemistry images of BrdU-positive cells in the 2D culture. Scale bar, 50 μm. (**d**) Percent of BrdU-positive cells. n = 3. At least 400 cells were counted at each cell density. (**e**) Cell viability was measured by trypan blue staining. (**f**) 2-NBDG uptake in the 2D culture was analyzed by confocal fluorescence microscopy. Cells were incubated with glucose and FBS-free medium for 24 h, after which 100 μM 2-NBDG was added, followed by incubation for 25 min at 37 °C. (**g**) Fluorescence intensity of 2-NBDG in the 2D culture. (**h**) 2-NBDG uptake in the 3D culture was analyzed by confocal fluorescence microscopy. (**i**) Fluorescence intensity of 2-NBDG in the 3D culture. (**j**) Re-growth of degraded spheroids in the 3D culture compared with that in the 2D culture. Cells were incubated for 4 days to form S500 spheroids and for the same period for the 2D culture. After 4 days, cell numbers in the 2D culture and spheroids were confirmed to be similar. Then, cells were again placed in the 2D culture at 4.2 × 10^3^ cells/cm^2^. Cells were counted by trypan blue staining. n = 3. Bars represent standard deviations (SD).

Furthermore, we investigated the effects of cell density and spheroid formation on cell dormancy. Cancer dormancy is characterized by no proliferation, no death, metabolic suppression, and recovery of active status. First, we assessed cell proliferation in both the 2D culture and 3D culture. Ki-67, a cell proliferation marker, was downregulated in a manner dependent on cell density and spheroid size (Fig. 2a,b and Supplementary Figures S1a,b). In contrast, p21, a regulator of cell cycle progression in the G1 and S phases, was upregulated in spheroids. Bromodeoxyuridine (BrdU) is a thymidine analog used for monitoring cell proliferation. BrdU-positive cells were decreased in the same manner (Fig. 2c,d). These results suggested that cell proliferation was suppressed at high cell density. However, little cell death was observed in spheroids, even at their maximum sizes (Fig. 2e). To investigate metabolic activity, we measured the uptake amount of 2-NBDG, a fluorescent glucose analog. 2-NBDG fluorescence decreased with cell density in the 2D culture and spheroids (Fig. 2f–i). The decreased proliferation was reversible; when the spheroids were reduced by pipetting and re-plated in a flat plate 2D culture, the cells demonstrated a recovery of the proliferation rate to control levels with a slight delay (Fig. 2j). Altogether, these results indicated that spheroids or cells cultured under a high cell density in 2D demonstrated dormancy. We accordingly used them as models for dormant cancer cells.

### PpIX accumulation increases in dormant cancer cells

**Figure 3:**
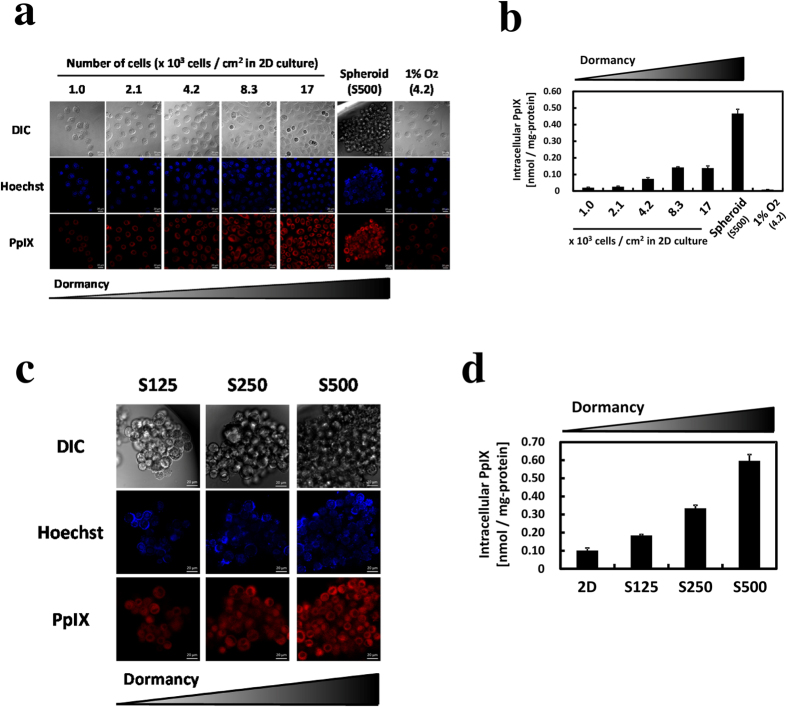
The effect of cell dormancy on PpIX accumulation. After incubation for 3 days, cells were incubated for 24 h with 1 mM ALA-containing medium. (**a**) Confocal laser scanning microscopy images of differential interference contrast, Hoechst 33342 for nuclei and PpIX. Scale bar, 20 μm. (**b**) PpIX accumulations under different cell density conditions in the 2D culture were measured by HPLC. n = 3. (**c**) Confocal laser scanning microscopy images of different sizes of spheroids. Scale bar, 20 μm. (**d**) PpIX accumulation at different spheroid sizes was measured by HPLC. n = 3. Bars represent standard deviation (SD).

To investigate the effect of cell dormancy on PpIX accumulation after ALA treatment, we evaluated the accumulation under different cell density conditions in the 2D culture. PpIX accumulation revealed a positive relationship with cell density (Fig. 3a,b). The PpIX amount increased 23-fold between the lowest cell density in the 2D culture and S500 spheroids. In contrast, PpIX accumulation decreased under hypoxic conditions. Thus, hypoxia was not involved with intracellular PpIX increase in dormant cancer cells. Similarly, PpIX accumulation revealed a positive relationship with spheroid size (Fig. 3c,d). These changes were inferred to have been caused by cell dormancy. Altogether, these results suggest that PpIX accumulation increases with cell dormancy under normoxic conditions.

### ALA-PDT cytotoxicity was induced in dormant cancer cells

**Figure 4:**
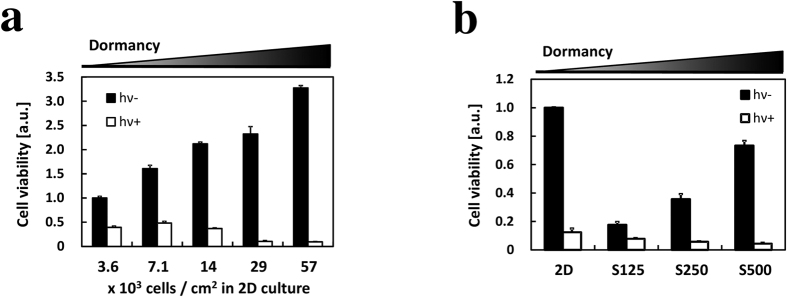
Effect of ALA-PDT on cell viability. Cells were incubated with 1 mM ALA in a complete medium for 24 h and then exposed to 1080 mJ/cm^2^ light for 5 min. Cell viability was determined on the next day by the MTT assay after treatment. (**a**) ALA-PDT in the 2D culture. Cells were incubated for 1 day before ALA treatment. n = 6. (**b**) ALA-PDT in spheroids. Cells were incubated for 3 days before ALA treatment. The cell density of “2D” is the same as that of “S500”. n = 3. Bars represent standard deviation (SD) (Table 1) Percentage cell viability in the 2D culture. (Table 2) Percentage cell viability in spheroids.

ALA-PDT can lead to cell death via the necrosis or apoptosis pathway and is a highly effective form of therapy for treating superficial basal cell carcinomas. We investigated ALA-PDT cytotoxicity under different cell-dormancy conditions. Cell viability in the 2D culture was reduced by ALA-PDT in a cell density-dependent manner (Fig. 4a and Table 1). Cell viability of spheroids was also reduced in a size-dependent manner (Fig. 4b and Table 2). These results are in good agreement with the increase in PpIX accumulation (Fig. 3b,d). They indicate that ALA-PDT cytotoxicity was induced by the increase in PpIX accumulation in a cell dormancy-dependent manner.

### PEPT1 and ABCB6 were upregulated depending on cell dormancy, whereas ABCG2 was downregulated

**Figure 5:**
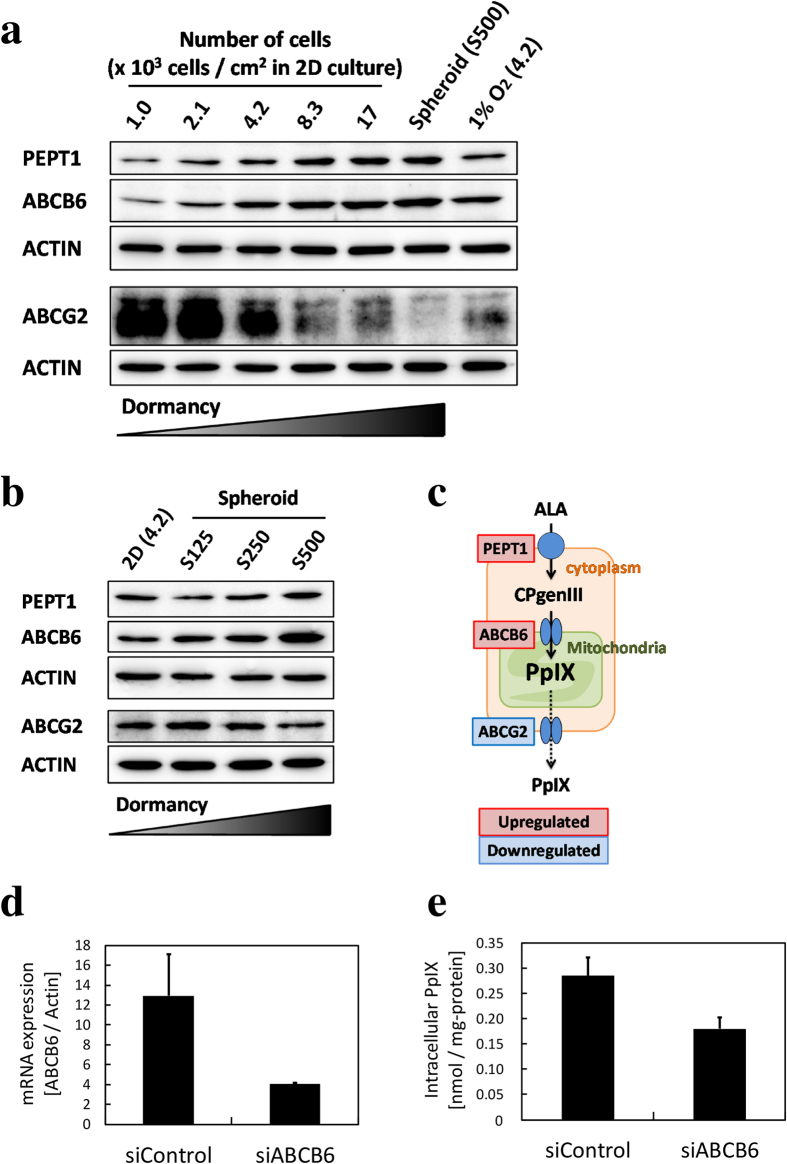
The protein expressions of PEPT1, ABCB6, and ABCG2 were detected by Western blotting. No cells were incubated with 1 mM ALA. (**a**) Expression at different cell density in the 2D culture. (**b**) Expression with different sizes of spheroids. (**c**) Regulation of transporters involved in porphyrin metabolism under high-cell-density 2D culture or in spheroids. (**d**) Expression of ABCB6 mRNA after incubation with siRNA in the high cell density-2D cultured cells. (**e**) PpIX accumulation after incubation with siRNA in the high cell density-2D cultured cells. CPgenIII: coproporphyrinogen III.

We assessed the protein expression of transporters involved in porphyrin metabolism. In our previous studies, we demonstrated that PEPT1, ABCB6, and ABCG2 were key players in regulating intracellular PpIX after ALA treatment. In summary, PEPT1 overexpression or ABCG2 suppressing caused increase of intracellular PpIX accumulation^24^. Other studies showed that ABCB6 overexpression induced increase of PpIX^12^ and ABCB6 suppressing induced decrease of PpIX^25^ respectively. Therefore, we focused on these 3 transporters. PEPT1 and ABCB6 were upregulated in a cell density-dependent manner in the 2D culture and size-dependent manner in the 3D culture (Fig. 5a–c; Supplementary Figures S2a,b). Furthermore, to examine the contribution of ABCB6 on dormant cancer cells, we used RNAi technology to suppress the expression of ABCB6 in the high cell density-2D cultured cells (Fig. 5d,e). The expressions of ABCB6 were suppressed by siRNA (Fig. 5d), and led to decreased PpIX accumulation (Fig. 5e). Since siABCB6 reduced PpIX accumulation in dormant cancer cells, it is suggested that increased expression of ABCB6 contributed to PpIX accumulation increase in dormant cancer cells. These results strongly suggest that ABCB6 is upregulated and affected PpIX accumulation in dormant cancer cells. In contrast, ABCG2 was downregulated in the same manner. Expression of neither hypoxia-inducible factors (HIF)-1α nor -2α was observed in S500, which was the maximum size of spheroids (Supplementary Figure S2c). These data also suggest 3D spheroids didn’t have the hypoxic area. mRNA expression of vascular endothelial growth factor (VEGF) was upregulated in a cell density-dependent manner (Supplementary Figure S2d). Altogether, these results suggest that cell dormancy affects not only one but multiple genes to enhance porphyrin production.

### PpIX accumulation increased in proliferation-inhibited dormant-like cancer cells

**Figure 6:**
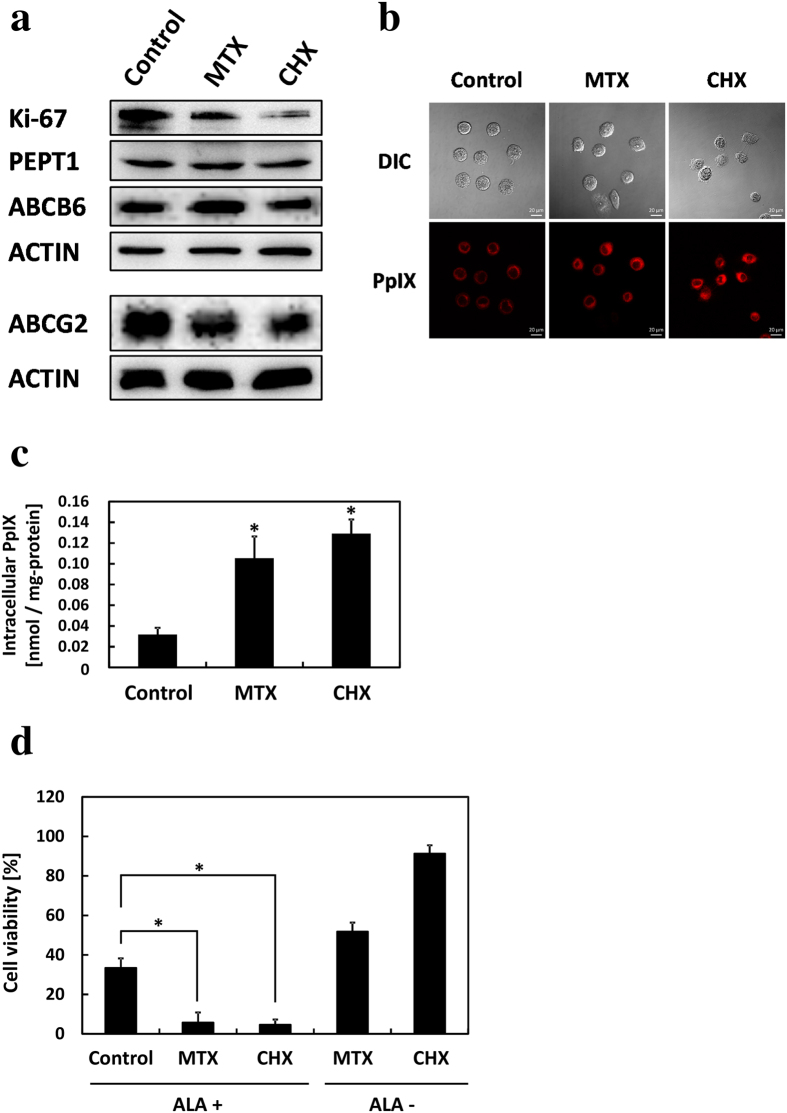
Porphyrin metabolism changed in cancer cells on addition of methotrexate (MTX) or cycloheximide (CHX). MTX was cultured with 10 μM and CHX was cultured with 10 μg/ml for 48 h. (**a**) Protein expression was detected by Western blotting. (**b**) Confocal laser scanning microscopy images of PpIX accumulation. Scale bar, 20 μm. (**c**) PpIX accumulation was measured by HPLC. n = 3. *p < 0.005, compared with control. (**d**) Effect of ALA-PDT on cell viability. Cells were incubated with 1 mM ALA for 24 h and then exposed to 1080 mJ/cm^2^ light for 5 min. Cell viability was determined with trypan blue. Cell viability was normalized by untreated control samples. n = 3. *p < 0.003. All bars represent standard deviation (SD).

In the previous section, we revealed that PpIX accumulation increased and cancer cell proliferation decreased in a cell dormancy-dependent manner. We accordingly hypothesized that PpIX accumulation is increased in proliferation-inhibited dormant-like cancer cells. To test this hypothesis, cells were cultured with methotrexate (MTX) or cycloheximide (CHX), which are reported to be G0/G1-phase arrestors for PC-3 cells^26^. Cell density was prepared similarly between control and G0/G1-phase arrestors (Supplementary Figure S3a). Ki-67 was downregulated by drug additions (Fig. 6a and Supplementary Figure S3b). Expression levels of PEPT1 and ABCB6 were not changed; however, ABCG2 was downregulated by both the drugs. PpIX accumulation was increased approximately 4-fold by drug additions (Fig. 6b,c). Next, we investigated ALA-PDT cytotoxicity after visible light irradiation under these conditions. Cell viability was markedly reduced, which is a result in good agreement with the increased PpIX accumulation (Fig. 6d). However, remarkable cell deaths were not observed in ALA- group. Altogether, these results indicated that ALA-PDT cytotoxicity increased in dormant-like cancer cells.

## Discussion

In this study, we first demonstrated that cancer cells exhibited dormancy, including the decrease of cell proliferation, dependent on cell density and spheroid size. Cell dormancy was characterized by no proliferation, no death, metabolic suppression, and recovery of active status. Second, we demonstrated an increase of PpIX accumulation after ALA treatment with the same dependency. We infer that PpIX accumulation increases with cell dormancy. Moreover, ALA-PDT cytotoxicity was induced and transporters involved in porphyrin metabolism were regulated in a cell dormancy-dependent manner in both the 2D culture and spheroids. PpIX accumulation was increased by incubation with ALA and G0/G1-phase arrestors and ALA-PDT cytotoxicity was also induced. To the best of our knowledge, this is the first study to demonstrate that dormant cancer cells accumulate high PpIX levels and are sensitive to ALA-PDT.

Many methods of 3D multicellular spheroid culture for biomedical research have been reported^19^. For example, matrigel culture, spinner flasks, and hanging drop are well-known methods for spheroid culture. In this study, we used the EZSPHERE 3D culture plate to form spheroids. This culture plate has been particularly used for embryonic stem cell research, and this is the first report to use the culture plate to form cancer spheroids^27^,^28^,^29^. This culture plate has two advantages. First, it allows the development of uniformly sized spheroids and the control of spheroid size by change in the number of cells seeded in the plate (Fig. 1c). Second, the culture medium can be exchanged with other media with the preservation of the spheroid 3D structure so that spheroids in the plate permit PpIX accumulation analysis by confocal fluorescence microscopic imaging (Fig. 3a,c). Finally, spheroids formed in the plate demonstrate the same phenotypes as described in previous reports^15^,^22^: inhibition of cell proliferation and VEGF upregulation (Supplementary Figure S2d).

Although understanding of porphyrin and heme biosynthesis has helped to advance the field of ALA-PDD and PDT, no well-defined mechanism of PpIX accumulation in cancer cells after ALA administration is known. In some studies, correlation between cell density and PpIX accumulation in the 2D culture has been suggested^30^,^31^. Georgakoudi *et al*. and Moan *et al*. reported that high-density populations produce substantially higher amounts of PpIX per cell than low-density populations but did not suggest a clear mechanism. In other studies, correlation in the 2D culture between cell proliferation and PpIX accumulation has been found^30^,^32^,^33^. Moan *et al*. have reported that the only cell cycle dependence of PpIX synthesis appears to be simple volume dependence in WiDr (human colon adenocarcinoma) cells. Given that cells in the G2 and M phases produce 1.9 times as much PpIX as cells in the G1 phase, the increase of PpIX accumulation could be explained by cell volume. In this study, PpIX accumulation also demonstrated a positive relationship with cell density in the 2D culture, and this relationship was observed even in the 3D culture (Fig. 3b,d). PpIX accumulation analysis of cells incubated with ALA and G0/G1-phase arrestors (methotrexate or cycloheximide) revealed that the PpIX amount was increased in PC-3 cells, although cell volume was unchanged (Fig. 6b). Thus, the effects of cell density on PpIX production may be cell line-dependent.

Gene expressions were dramatically altered in a cell dormancy-dependent manner. To the best of our knowledge, this is the first report to demonstrate that PEPT1, ABCB6, and ABCG2 expression levels are regulated by cell density or dormancy. ABCG2 was upregulated in gastric and ovarian cancer spheroids^34^,^35^. In our study, ABCG2 was downregulated, and one of the causes was suppression of cell proliferation. Because cells treated with G0/G1-phase arrestors expressed less ABCG2 (Fig. 6a). However, the final reason for PEPT1 and ABCB6 upregulation is unknown. In our previous study and others, PEPT1 and ABCB6 were upregulated under hypoxic conditions^11^,^36^,^37^,^38^. However, we could not detect HIF expression in the 2D or 3D culture (Supplementary Figure S2c). Cells treated with CoCl_2_ or DMOG, which are known as HIF inducers, did not demonstrate increased PpIX accumulation (data not shown). Furthermore, coproporphyrin III, which is eliminated from cells under hypoxic conditions after ALA treatment, was not detected in spheroids^11^. These results suggest that hypoxia is not a critical factor in PpIX accumulation increase. However, TNF-α and IFN-γ increase PEPT1 expression and activity^39^. Transporter expression may be upregulated by cytokine concentration in a cell density-dependent manner.

Recently, cancer spheroids have been reported to reflect heterogeneous tumor tissues more accurately in the 3D culture than in the 2D culture. Gene expression in spheroids is heterogeneous owing to exposure to oxygen and nutrients and to other physical and chemical stresses in different microenvironments^40^,^41^. Ki-67 positive cells were present at edges of spheroids, and the cells in the center were dormant^42^,^43^,^44^. We demonstrated that dormant cancer cells tended to accumulate high PpIX. Accordingly, we expected that PpIX levels in the cells in the center would be high and those at the edges would be low. However, we observed that the cells at the edges accumulated high PpIX and the cells in the center accumulated low PpIX (Fig. 3c). This observation may reflect two points. One point is the heterogeneity of micro-environmental ALA concentrations, given that cells at the edges readily take up ALA from the medium. The other point is the heterogeneity of cancer dormancy. In other words, the cells at the edges might be more dormant than those in the center in our spheroids which made by EZSPHERE. The distinct reason of PpIX heterogeneity in spheroids is unknown. On the other hand, PpIX levels of cells were also heterogeneous in the 2D culture. The PpIX level changed more than 2-fold between high- and low-PpIX-accumulating cells in the same culture dish (Fig. 3a). This phenomenon is in good agreement with BrdU-positive heterogeneity.

The results of this study indicate that ALA-based treatment would be an innovative therapy for dormant cancer cells, which are relatively insensitive to most chemotherapeutic drugs and radiation. In addition, these cells can cause tumor recurrence when they re-enter the cell cycle^13^,^15^,^16^. Accordingly, much research has been attempted to improve treatments for resistance. In this study, we demonstrated that dormant cancer cells tended to accumulate high PpIX and had higher sensitivity to ALA-PDT than non-dormant cells (Fig. 4a,b). Moreover, ALA-PDT cytotoxicity to non-dormant cells could be enhanced by combination with G0/G1-phase arrestors (Fig. 6d). In previous studies, combination with methotrexate (MTX) enhanced ALA-PDT in skin carcinoma and prostate cancer^45^,^46^. In brief, these authors argued that PpIX accumulation was induced by MTX and that one of the causes was the upregulation of coproporphyrinogen oxidase. From previous reports and the increase of PpIX accumulation in dormant cancer cells in this study, we propose that ALA-based diagnosis and therapy will be effective approaches for dormant cancer cells. However, the reason for high PpIX accumulation in those cells remains unclear. Further study of the PpIX accumulation mechanism in dormant cancer cells should contribute to ALA-based treatment.

## Methods

### Cells and cell cultures

The human prostate cancer cell line PC-3 (provided by Dr. Inoue, Kochi University, Kochi, Japan) was maintained in an RPMI-1640 medium supplemented with 10% (v/v) FBS and 1% (v/v) ABAM. Cells were maintained under 5% CO_2_ gas at 37 °C. Cell culture under hypoxic conditions (1% oxygen) was performed with an AnaeroPack-Kenki 5% (Mitsubishi Gas Chemical Co., Tokyo, Japan).

### 3D cell culture

EZSPHERE 3D cell culture plates were obtained from AGC Techno Glass Co., Ltd. (Tokyo, Japan) and used to culture cancer spheroids. Totals of 5.00 × 10^5^ cells for S500, 2.50 × 10^5^ cells for S250, and 1.25 × 10^5^ cells for S125 were seeded with 3 ml medium on every 35-mm dish. After 2 days, 1 ml of old medium was carefully replaced with new medium. Four days after seeding, 2,300 spheroids were formed in each dish.

### Biochemicals

ALA hydrochloride was purchased from Cosmo Oil Co., Ltd. (Tokyo, Japan). RPMI-1640 medium, antibiotic antimycotic solution (ABAM), and protease inhibitor cocktail were purchased from Nacalai Tesque (Kyoto, Japan). Fetal bovine serum (FBS) was purchased from Invitrogen (Carlsbad, United States). Hoechst 33342 was purchased from Sigma-Aldrich (St. Louis, United States). BrdU kit was purchased from Roche (Mannheim, Germany). 2-NBDG was purchased from Cayman Chemical (Michigan, United States). All other chemicals used were of analytical grade.

### HPLC analysis of PpIX

Cells were incubated with 1 mM ALA under 5% CO_2_ at 37 °C in normoxia (21% oxygen) or hypoxia (1% oxygen) in the dark for 24 h. High-performance liquid chromatography (HPLC) analysis was performed as previously described with some modifications^11^,^47^. Cells were washed and lysed using 0.1 M NaOH, and lysates were extracted by addition of three volumes of DMF/2-propanol (100:1, v/v). Mixtures were then centrifuged to remove proteins, and supernatants were incubated at room temperature in the dark for 1 day. Then, 100 μL aliquots were collected, and porphyrins were separated using an HPLC system (Type Prominence, Shimadzu, Kyoto, Japan) equipped with a reversed-phase C18 column (CAPCELLPAK, C18, SG300, 5 μm, 4.6 mm × 250 mm; SHISEIDO, Tokyo, Japan) maintained at 40 °C. Elution solvent A contained 1 M ammonium acetate and 12.5% acetonitrile (pH 5.2), and solvent B contained 50 mM ammonium acetate and 80% acetonitrile (pH 5.2). Porphyrins were eluted with solvent A for 5 min, with a linear gradient of solvent B (0–100%) for 25 min and then with solvent B for 10 min. Flow was maintained at a constant rate of 1.0 mL/min, and porphyrins were continuously detected using a fluorospectrometer (excitation at 404 nm, detection at 624 nm). Porphyrin concentrations were estimated using calibration curves from porphyrin standards.

### Western blot analyses

Western blot analyses were performed as previously described^48^. Primary antibodies used against Ki-67 (ab15580, 1:1000 dilution) and PEPT1 (ab55936, 1:200 dilution) were purchased from Abcam (Cambridge, Great Britain); p21 (sc-397, 1:200 dilution) from Santa Cruz Biotechnology (Texas, United States); ABCB6 (600-401-945, 1:500 dilution) from Rockland (Limerick, Ireland); ABCG2 (BXP-21, 1:200 dilution) from Convance Research Products (Emeryville, Canada); HIF-1α (AF1935, 1:400 dilution) from R&D Systems (Minneapolis, Mongolia); HIF-2α (NB100-122, 1:500 dilution) from Novus Biologicals (Littleton, United States); actin (691001, 1:500 dilution) from MP Biomedicals (Santa Ana, United States). For the second antibody, we used anti-mouse and anti-rabbit IgG HRP-conjugated antibody (1:3000 dilution) from Cell Signaling Technology (Beverly, United States).

### Confocal fluorescence microscopic imaging

Cells were implanted in flat normal cell-culture dishes for the 2D culture or in EZSPHERE for the 3D culture and incubated for 3 days. After cancer spheroid formation, cells were incubated with 1 mM ALA under 5% CO_2_ at 37 °C in normoxia (21% oxygen) or hypoxia (1% oxygen) in the dark for 24 h. Then, the ALA-containing medium was removed, and cells were incubated with 1 μM Hoechst 33342-containing medium for 20 min. Cells were rinsed twice with PBS and incubated in HBSS for confocal imaging. A Zeiss LSM 780 upright laser scanning confocal microscope (Carl Zeiss SAS, Jena, Germany) was used for live-cell imaging. The excitation was set at a wavelength of 405 nm for PpIX and Hoechst, 488 nm for BrdU and 458 nm for 2-NBDG. The emission was set at 620–700 nm for PpIX, 440–500 nm for Hoechst, 493–630 nm for BrdU, and 500–600 nm for 2-NBDG. Laser illumination was set at 2.0% power for all. Images were acquired using a 40× water immersion lens for PpIX, Hoechst, and 2-NBDG, and a 20× lens for BrdU. The images were analyzed with the ZEN software package of Zeiss.

### Establishment of cancer cell lines transiently suppressing endogenous ABCB6 protein

Small interfering RNA (siRNA) oligonucleotides and negative control scrambled oligonucleotides were custom synthesized by Dharmacon (Lafeyette, CO, USA)^11^ with the following sequences:

siRNA (ABCB6)

sense 5′-CUGUUUCGCUUCUACGACAtt-3′

antisense 5′-UGUCGUAGAAGCGAAACAGtt-3′

Oligonucleotides were transfected into cells (16 × 10^3^ cells/cm^2^ in 2D culture) at a final concentration of 5 nM using DharmaFECTTM 4 (Dharmacon) according to the manufacturer’s instructions. Cells were further incubated with 1 mM ALA for 24 h. mRNA and PpIX were extracted and measured as previously described^11^.

### Exposure of the cells to light-emitting diode (LED)

Cells were incubated with 1 mM ALA under 5% CO_2_ at 37 °C for 24 h. Cells were then exposed to LED irradiation for 5 min (630 nm, 1080 mJ/cm^2^) by placement of the plate under an LED irradiation unit (provided by SBI Pharma CO., Ltd., Tokyo, Japan) as previously described^24^. This irradiation condition is almost equivalent to the clinical irradiation conditions. Cells were further incubated in the dark under 5% CO_2_ at 37 °C for 24 h. Cell viability was then measured by the MTT assay or trypan blue staining as previously described^49^.

## Additional Information

**How to cite this article**: Nakayama, T. *et al*. Dormant cancer cells accumulate high protoporphyrin IX levels and are sensitive to 5-aminolevulinic acid-based photodynamic therapy. *Sci. Rep.* **6**, 36478; doi: 10.1038/srep36478 (2016).

**Publisher’s note**: Springer Nature remains neutral with regard to jurisdictional claims in published maps and institutional affiliations.

## Supplementary Material

Supplementary Information

## Figures and Tables

**Table 1:** Cell viability in the 2D culture.

	x 10^3^ cells/cm^2^ in 2D culture				
	3.6	7.1	14	29	57
**Cell viability [%]**	39.2	30.0	17.4	5.0	2.9

**Table 2:** Cell viability of spheroids.

	2D*	S125	S250	S500
**Cell viability [%]**	12.4	44.6	16.1	6.0

^*^The cell density of “2D” is the same as that of “S500”.
