# Supplementary material for: Dormant cancer cells accumulate high protoporphyrin IX levels and are sensitive to 5-aminolevulinic acid-based photodynamic therapy
Source: Sci Rep. 2016 Nov 18;6:36478. doi: 10.1038/srep36478 (PMC5114660; doi:10.1038/srep36478)
Supplement: Supplementary Information [file srep36478-s1.pdf]

Supplementary Information

Dormant cancer cells accumulate high protoporphyrin IX levels and are sensitive to 5-aminolevulinic acid-based photodynamic therapy

Taku Nakayama[1], Shimpei Otsuka[1], Tatsuya Kobayashi[1], Hodaka Okajima[1], Kentaro Matsumoto[1], Yuichiro Hagiya[1], Keiji Inoue[2], Taro Shuin[2], Motowo Nakajima[3], Tohru Tanaka[3], Shun-ichiro Ogura[1*].

1. Graduate School of Bioscience and Biotechnology, Tokyo Institute of Technology, 4259 B47, Nagatsuta-cho,Midori-ku, Yokohama 226-8501, Japan.
2. Department of Urology, Kochi Medical School, Kohasu, Okocho, Nankoku, Kochi, 783-8505, Japan.
3. SBI Pharmaceuticals Co., Ltd., Izumi Garden Tower 20F, 1-6-1, Roppongi, Minato-ku, Tokyo 106-6020, Japan.

*Corresponding author: Shun-ichiro Ogura

Department of Bioengineering, Graduate School of Bioscience and Biotechnology, Tokyo Institute of Technology, 4259 B47, Nagatsuta-cho,Midori-ku, Yokohama 226-8501, Japan.

Tel: +81-045-924-5845; Fax: +81-045-924-5845; Email: sogura@bio.titech.ac.jp

a

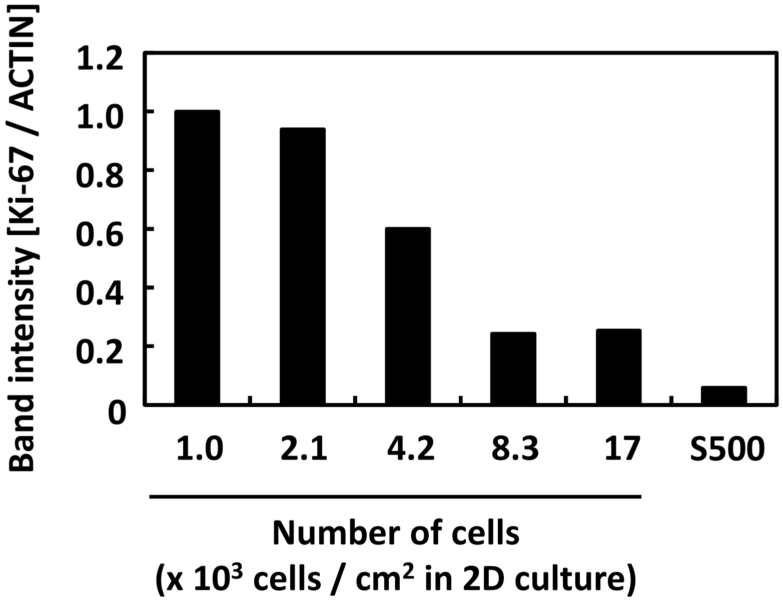

b

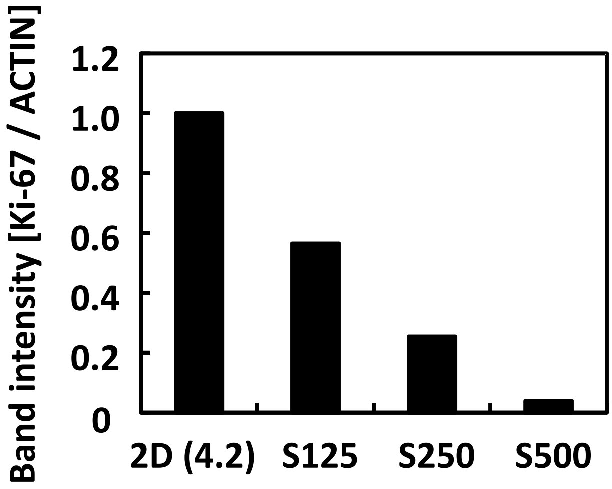

Supplementary Figure S1

(a) Band intensity of Ki-67 in 2D culture. (b) Band intensity of Ki-67 in spheroids.

a

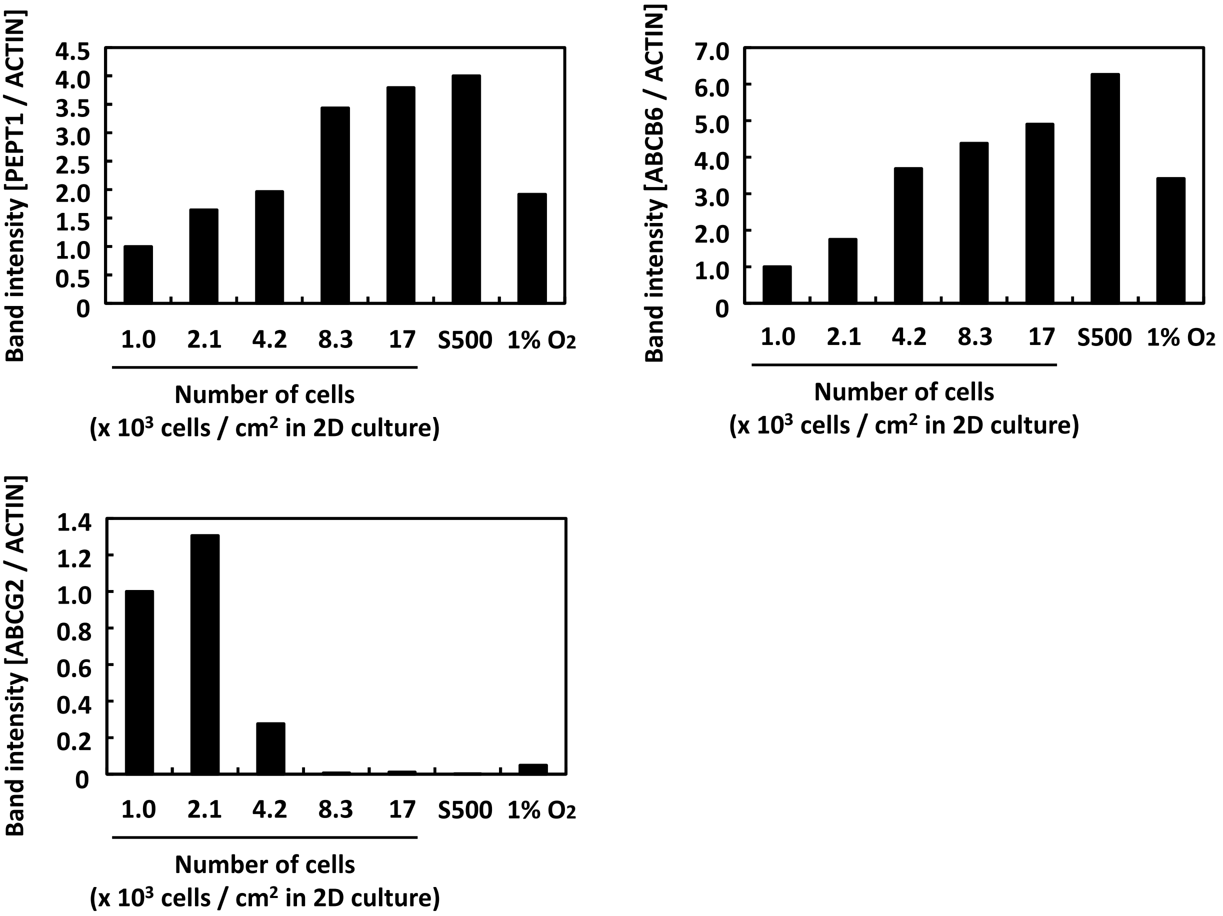

Band intensity [PEPT1 / ACTIN]
4.5
4.0
3.5
3.0
2.5
2.0
1.5
1.0
0.5
0
1.0 2.1 4.2 8.3 17 S500 1% O2
Number of cells
(x 10³ cells / cm² in 2D culture)
Band intensity [ABCB6 / ACTIN]
7.0
6.0
5.0
4.0
3.0
2.0
1.0
0
1.0 2.1 4.2 8.3 17 S500 1% O2
Number of cells
(x 10³ cells / cm² in 2D culture)
Band intensity [ABCG2 / ACTIN]
1.4
1.2
1.0
0.8
0.6
0.4
0.2
0
1.0 2.1 4.2 8.3 17 S500 1% O2
Number of cells
(x 10³ cells / cm² in 2D culture)

b

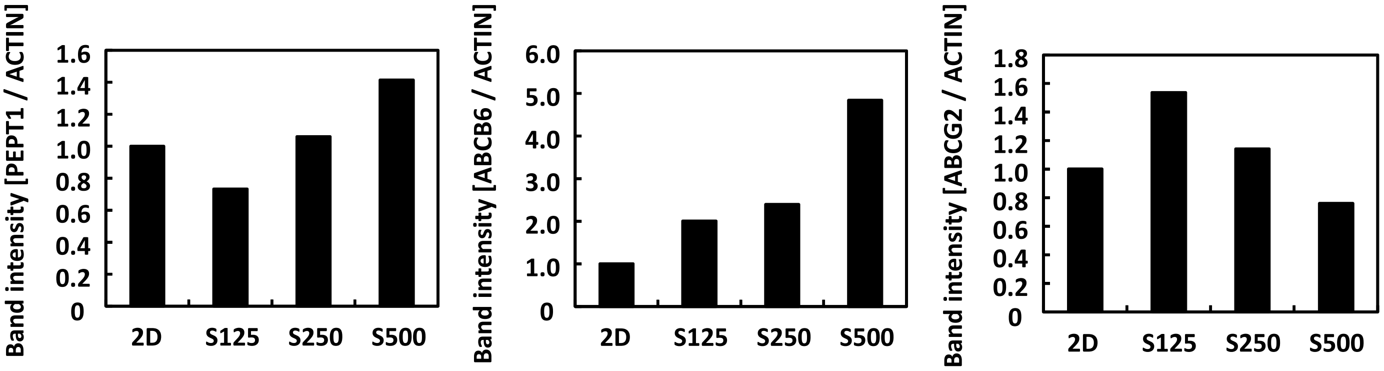

Band intensity [PEPT1 / ACTIN]
1.6
1.4
1.2
1.0
0.8
0.6
0.4
0.2
0
2D S125 S250 S500
Band intensity [ABCB6 / ACTIN]
6.0
5.0
4.0
3.0
2.0
1.0
0
2D S125 S250 S500
Band intensity [ABCG2 / ACTIN]
1.8
1.6
1.4
1.2
1.0
0.8
0.6
0.4
0.2
0
2D S125 S250 S500

c

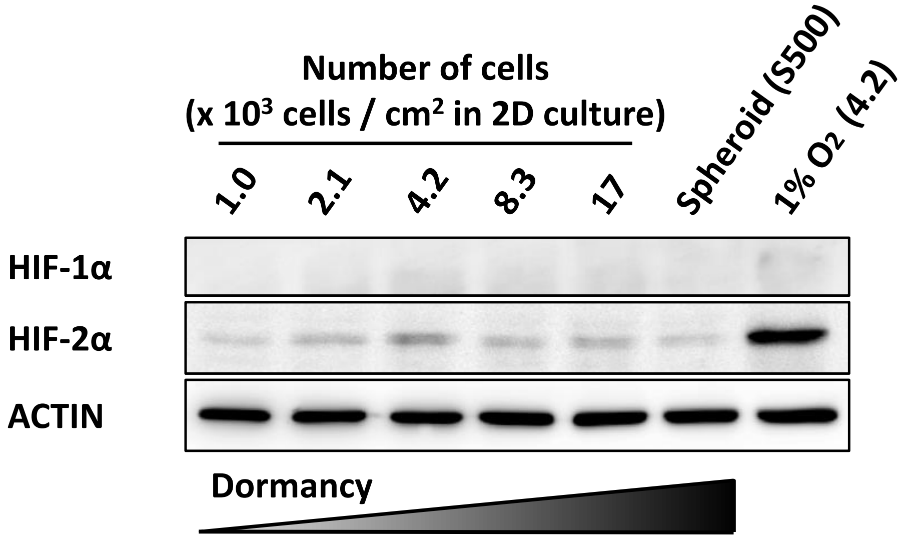

d

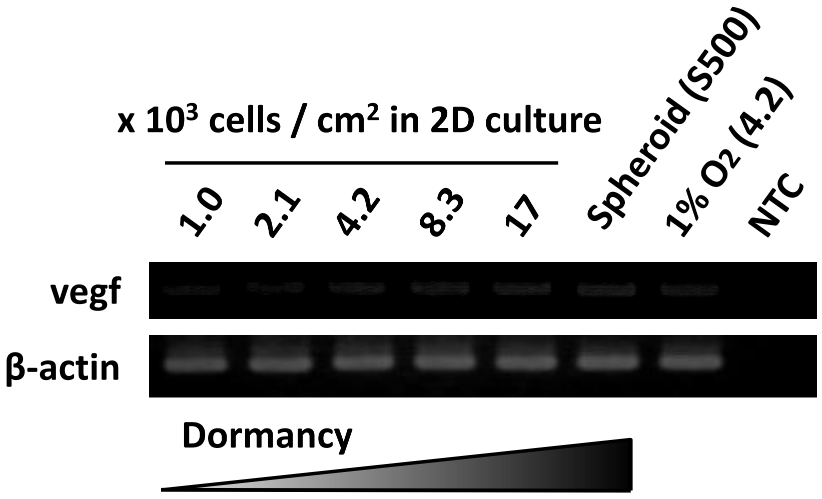

Supplementary Figure 2

(a) Band intensity of PEPT1, ABCB6, and ABCG2 in the 2D culture. (b) Band intensity of PEPT1, ABCB6, and ABCG2 in spheroids. (c) HIF protein expression at different cell densities was detected by Western blotting. (d) VEGF mRNA expression at different cell densities was detected by RT-PCR. NTC: no template control

a

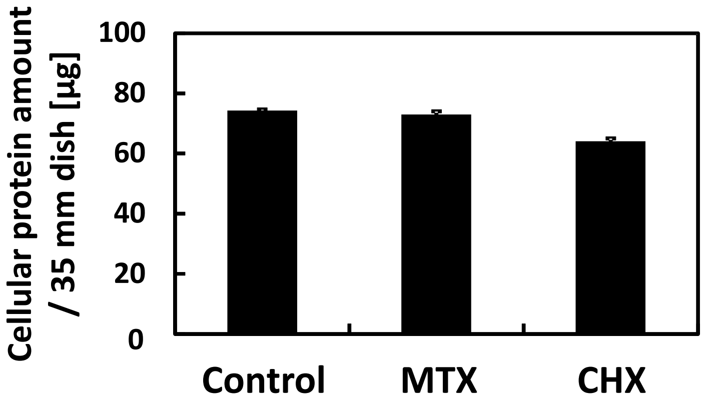

b

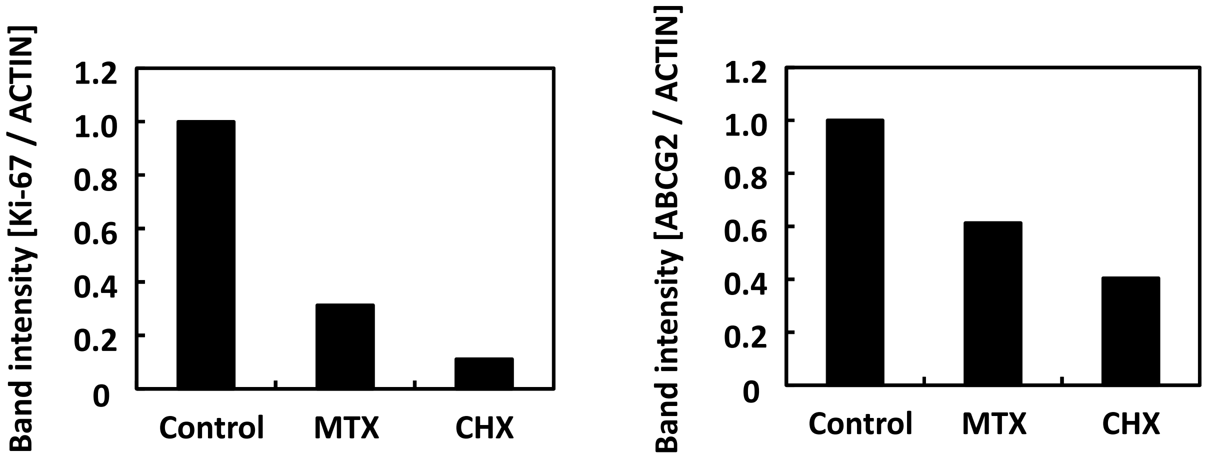

Supplementary Figure 3

(a) Cellular protein amount per 35-mm dish. Cells were incubated with methotrexate or cycloheximide for 48 h. Protein concentration was measured by Bradford assay. n = 3. (b) Band intensity of Ki-67 and ABCG2.
